# Adherence to Bisphosphonates among People Admitted to an Orthopaedic and Geriatric Ward at a University Hospital in Sweden

**DOI:** 10.3390/pharmacy6010020

**Published:** 2018-02-28

**Authors:** Linnea Abramsson, Maria Gustafsson

**Affiliations:** Department of Pharmacology and Clinical Neuroscience, Umeå University, SE-90187 Umeå, Sweden; linnea-a@hotmail.com

**Keywords:** adherence, bisphosphonates, elderly

## Abstract

Oral bisphosphonates are the first choice of therapy to reduce the risk of osteoporotic fractures. These medications have generally poor oral bioavailability, which may further be reduced by concomitant intake of certain foods and drugs; therefore, it is vital to follow specific instructions. The aim with this study was to assess general adherence to oral bisphosphonates and adherence to specific administration instructions among people admitted to two wards at Umeå University hospital in Sweden. This interview study focuses on elderly patients living at home and prescribed oral bisphosphonates. Invited were 27 patients admitted to an orthopaedic ward and a geriatric ward during the period 28 March 2017 and 5 December 2017. In total, 21 patients were interviewed regarding their adherence to oral bisphosphonates. Out of 21 patients, 13 (62%) were considered non-adherent. The most common reason was calcium intake less than 2 h after oral administration of bisphosphonate (54%). The number of regularly prescribed drugs was significantly higher among patients rated non-adherent to bisphosphonates compared to those rated adherent (*p =* 0.004). Adherence to bisphosphonates administration instruction among elderly people living at home was limited. More research is needed to confirm these results and to investigate the reasons for non-adherence and how adherence to bisphosphonates can be improved.

## 1. Introduction

Osteoporosis is a systemic disease, characterized by low bone mass and structural deterioration of bone tissue, leading to an increased risk of fractures without significant trauma. Osteoporosis causes substantial morbidity and mortality and the incidence of osteoporotic fractures is expected to increase worldwide as a consequence of aging populations [1]. The disease can be divided into primary osteoporosis (for example postmenopausal osteoporosis), and secondary osteoporosis. Secondary osteoporosis is caused by a number of different diseases and may also be associated with drug use such as glucocorticoids [1]. 

Different drugs are used to prevent osteoporosis and to reduce the risk of osteoporotic fractures. In Sweden, oral bisphosphonates such as alendronic acid are nationally recommended [2]. Bisphosphonates attach to hydroxyapatite binding sites on bony surfaces, especially at sites of active resorption. Osteoclast activity and bone absorption is hampered in the presences of bisphosphonates. Furthermore, osteoclast activity is inhibited by decreased osteoclast progenitor development and increased osteoclast apoptosis [3]. One of the main problems with bisphosphonates is very low oral bioavailability, only approximately 1–7% of the administered dose [3], and risk of further reduction when taken together with certain foods and medications. This poses specific requirements on the patients. According to pharmaceutical specialties in Sweden [4] and Janusmed interactions [5], an optimum uptake is only obtained if specific instructions are followed. Maximum uptake is achieved if bisphosphonates are taken with a certain amount water on an empty stomach first thing in the morning. After administration, the patient should stand upright for at least 30–60 min and refrain from consuming food, drink, medications, or supplements for at least 30 min [4]. Furthermore, bisphosphonates can form complexes with a number of divalent cations such as magnesium and calcium, as well as iron supplements, which may further reduce absorption of bisphosphonates, and should be avoided during at least two hours after bisphosphonate intake [5].

Adherence with bisphosphonates among patients with osteoporosis is important since a high adherence has found to be associated with lower fracture incidence [6]. Adherence to osteoporosis medicines has however proven to be limited. For example, in one study conducted in Estonia where initiation, implementation, and persistence to these medications were found to be low [7].

Adherence to specific administration instructions to bisphosphonates has received limited attention in medical literature. The aim with this study was to assess general adherence to oral bisphosphonates and adherence to specific administration instructions among elderly people admitted to an orthopaedic ward and geriatric ward at Umeå University Hospital, Sweden. 

## 2. Materials and Methods

### 2.1. Material and Procedures

This interview study was conducted at the orthopaedic and the geriatric ward at Umeå University hospital, Sweden. Invited to participate were patients admitted to the two wards during the period 5 June 2017 and 1 July 2017, and to the orthopaedic ward during the period 3 March 2017 and 5 December 2017, living at home with oral bisphosphonates prescribed before admission. The patients were included subsequently during these periods. The interviews were performed at the wards, at the patients’ bedside.

The medical history of study participants was retrieved from the medical journals. Patient background information included age, gender, medications (except as drugs “as needed” and topical administrated drugs), indication for bisphosphonates, cause of hospitalization and number of months being prescribed bisphosphonate. In the interviews, the following questions were asked: time of the day for bisphosphonate intake; bisphosphonate intake in relation to their other medications and food; how the patients swallow the bisphosphonates (with water or other beverages); how often the patients forget to take the bisphosphonates (always, often, sometimes, rarely never, never); and finally if the patients get help with their medication at home.

### 2.2. Definition of Adherence to Bisphosphonates

Based on instructions from pharmaceutical specialties in Sweden [4] and Janusmed interactions [5], a patient defined as adherent was required to: (I) wait for at least 30 min after bisphosphonate intake before intake of food or other medications; (II) avoid intake of interacting drugs during at least two hours after bisphosphonate intake; (III) swallow the bisphosphonates with water, only, and avoid other beverages; (IV) “never” or “rarely never” forget to take the bisphosphonates. A patient was defined as adherent if all criteria (I–IV) were met. 

Since the amount of water can be hard for the patient to estimate, and an upright position is mainly recommended to avoid damage to the oesophagus and not to increase bioavailability, adherence to these administration instructions was not considered in the present study. On the other hand, an additional criterion for quantifying adherence to bisphosphonate intake was included in the study (never or rarely never forget to take the bisphosphonates).

### 2.3. Statistics

Different factors related to adherence and non-adherence, respectively, were investigated using the Pearson chi-square test and the independent sample *t*-test. A *p*-value of <0.05 was considered statistically significant. Statistical calculations were performed using the SPSS Statistics 23.

### 2.4. Ethics

The regional Ethics Review Board in Umeå approved the study (nr 2017/216-31 and 2017/319-32). 

## 3. Results

Between 28 March 2017, and 5 December 2017, 27 patients admitted to the orthopaedic and the geriatric ward and treated with oral bisphosphonates, were invited to participate in the study. Six patients declined participation. The remaining 21 patients were interviewed during their hospital stay. The average mean age of the patients was 75.2 years, the average mean number of medicines was 10.0 and the average mean numbers of years patients had bisphosphonate was 3.4 years (see Table 1).

According to the definition used in this study, 13 of the 21 interviewed patients (61.9%) were classified as non-adherent. The most common cause of non-adherence was calcium administration within 2 h of oral bisphosphonate intake (*n* = 7/13, 54%) (see Table 2). Two of these patients had dose-dispensed drugs (the drugs are dispensed into one unit for each dose occasion) and, in addition, assistance in drug administration by health care professionals. Intake of bisphosphonates together with food/other beverages than water and always/often/sometimes forget to take their bisphosphonates, were other causes for non-adherence. Moreover, two persons did not remember how they took their bisphosphonates and were therefore judged as non-adherent.

When comparing patients judged as non-adherent with patients considered adherent to bisphosphonates on a group base, there was no statistically significant difference between the groups except for the number of regularly prescribed drugs, which was significantly higher among patients judged non-adherent to bisphosphonates (*p =* 0.004) (see Table 1).

## 4. Discussion

This is a small study, and consequently, the results can only give an indication of patients’ level of adherence to bisphosphonates. However, because adherence to specific administration instructions to bisphosphonates has received limited attention, these results are still important to pay attention to. Of 21 patients, only 8 were adherent to bisphosphonates according to criteria for adherence used in this study. Specifically, the majority of patients failed to adhere to the recommendation not to take drugs potentially interfering with bisphosphonate absorption such as calcium during a period of 2 h after bisphosphonate intake. 

According to a recent comprehensive review, the overall prevalence of non-adherence among old people with polypharmacy living at home is expected to be high, between 6–55% [8]. The prevalence of non-adherence specifically to bisphosphonates in the present study was slightly higher (62%). We found that improper drug intake rather than medication persistence was the major adherence problem in the present material. Moreover, different definitions of adherence used in publications make comparability of prevalence numbers between the studies problematic [9,10,11,12,13,14,15,16,17,18]. A previous registry study performed in Sweden found that the persistence to treatment of osteoporosis was generally poor, 50% of patients terminated the treatment within a year. However, among patients who continued treatment with osteoporosis drugs, adherence was high [6]. In the present study, all patients had initiated bisphosphonate treatment and only two of the interviewed patients did not take their medication regularly. It appears thus that initiation of treatment and persistence is a minor problem in the present material. The adherence problems in this study were mainly associated with failures to follow the specific administration instructions. The most common reason for non-adherence was concomitant intake of calcium and bisphosphonate and neglecting the recommended time intervals between administration time points. Bisphosphonates should be prescribed with calcium and vitamin D supplements, but it is important that the patient is informed and follows the instructions to avoid calcium administration within two hours after oral intake of bisphosphonates to safeguard bioavailability. 

Two patients in the present study did not remember how they took their medications and had difficulties in understanding some of the interview questions. Considering they were normally living at home administrating their medications on their own, this raises issues regarding the general capability to proper drug administration. Even a small cognitive impairment has been proven to negatively affect adherence to medications [19]. Admittedly, instructions for proper administration of bisphosphonates are rather complex and require a high level of instructions by health care professionals and patient awareness [20]. According to guidelines, those individuals who have difficulty in complying with alendronic acid treatment should instead be treated with zolendronic acid (intravenous bisphosphonate) [5], and this is an option that perhaps should be used more often to assure effectiveness of osteoporosis prophylaxis [20]. 

In the present study, patients who were judged as non-adherent to bisphosphonates were generally treated with a larger number of medications compared to those not judged adherent in the present study. A larger study might have found more significant associations between adherence and different factors. Nevertheless, the result is in line with a recent review where medication adherence was negatively associated with a greater number of drugs among people living at home [8]. Polypharmacy may in general affect medication adherence, especially in elderly people [21]. Intuitively, higher medication adherence—as well as external help with medications—would be expected in patients having access to dose-dispensing systems [22]. However, since calcium and alendronate were prescribed concomitantly by the prescribers probably without proper administration instructions, no positive effects of dose dispensing systems and help with medication administration could be seen in our material. This could also be related to the low number of observations. However, in a large study on medication adherences in patients with diabetes from Switzerland, no impact of dose dispensing systems on medication adherence was observed [23]. These findings and observations in the present study may thus indicate that there should be an increased focus on proper information regarding oral bisphosphonates administration and this regards not only the patients, but also prescribers, home care staff, and relatives of the patients. 

This study has a number of limitations that need to be considered. Overall, the number of patients included in the present study was limited and patients were admitted to the hospital for different reasons, the great majority to an orthopaedic ward, and only one patient to a geriatric ward during the study period. Thus, the representativeness of the study population is low and the results should be interpreted carefully, as there is risk of bias and chance findings. Also, the chance to find statistically significant relationships is very low due to the limited number of observations, and the results should be interpreted with caution for that reason. In conclusion, this study can only give a rough estimate of patients’ level of adherence to bisphosphonates when living at home. Hospital admission in itself may impose stress on the patients impairing memory functions [24]. Nevertheless, our results are by-and-large in line with published literature and indicate a limited adherence among this group of patients prescribed bisphosphonates. More research is needed how adherence to bisphosphonates can be improved.

## 5. Conclusions

This small study found that adherence to bisphosphonates among elderly people living at home was poor and mostly related to improper administration. More research is needed to confirm these results and to investigate the reasons for non-adherence and how adherence to bisphosphonates can be improved.

## Figures and Tables

**Table 1:** Characteristics of study population and comparison between people adherent and non-adherent to bisphosphonates.

	Non-Adherent (*n* = 13)	Adherent (*n* = 8)	Total (*n* = 21)	*p*-Value
Women; *n* (%)	13	8	21 (100.0)	
Mean age ± SD	74.8 ± 15.6	75.9 ± 8.5	75.2 ± 13.1	0.857
Ward				0.421
Orthopaedic ward; *n* (%) Geriatric ward; *n* (%)	12 (92.3) 1 (7.7)	8 (100.0) 0 (0.0)	20 (95.2) 1 (4.8)	
Cause of admission *				0.604
Fractures; *n* (%)	5 (38.5)	4 (50.0)	9 (42.9)	
Other; *n* (%)	8 (61.5)	4 (50.0)	12 (57.1)	
Cognitive impairment ** *n* (%)	3 (14.3)	0 (0.0)	3 (14.3)	0.142
Number of regular medications mean ± SD	12.5 ± 5.3	5.9 ± 2.7	10.0 ± 5.5 (3–23)	0.004
Dose-dispensed drugs *n* (%)	3 (14.3)	0 (0.0)	3 (14.3)	0.142
Help with medications *n* (%)	4 (30.8)	1 (12.5)	5 (23.8)	0.340
Indications for bisphosphonates Confirmed osteoporosis; *n* (%) Glucocorticoid use; *n* (%)	9 (69.2) 4 (30.8)	7 (87.5) 1 (12.5)	16 (76.2) 5 (23.8)	0.340
Numbers of years with bisphosphonates mean ± SD	3.1 ± 2.6	4.0 ± 4.8	3.4 ± 3.5 (1–15)	0.619
Calcium intake; *n* (%)	13 (100.0)	7 (87.5)	20 (95.2)	0.191

* The most common fractures were femure fractures. Other causes of hospitalisation were for example pain, infections, and post-operative care. ** A patient was defined with cognitive impairment if this was stated in the medical record when the patient was admitted to the hospital.

**Table 2:** Reasons for non-adherence to bisphosphonates.

Patients non-adherent	*n* = 13
Calcium administrated within two hours; *n* (%)	7 (54.0)
Intake of bisphosphonates together with food/other beverages than water; *n* (%)	2 (15.4)
Always/often/sometimes forget to take their bisphosphonates; *n* (%)	2 (15.4)
Did not remember how they took their bisphosphonates; *n* (%)	2 (15.4)

## References

[1] Tarantino U., Iolascon G., Cianferotti L., Masi L., Marcucci G., Giusti F., Marini F., Parri S., Feola M., Rao C. (2017). Clinical guidelines for the prevention and treatment of osteoporosis: Summary statements and recommendations from the Italian Society for Orthopaedics and Traumatology. J. Orthop. Traumatol..

[2] The National Board of Health and Welfare National Guidelines for Musculoskeletal Diseases (2015). http://www.socialstyrelsen.se/nationellariktlinjerforrorelseorganenssjukdomar.

[3] Lin J.H. (1996). Bisphosphonates: A review of their pharmacokinetic properties. Bone.

[4] (2003). Alendronate. SPC. Fass (The Swedish Catalogue of Approved Medical Products).

[5] Stockholm County Council, the Health and Medical Care Administration Janusmed interactions. https://janusmed.sll.se/interaktioner.

[6] Landfeldt E., Strom O., Robbins S., Borgstrom F. (2012). Adherence to treatment of primary osteoporosis and its association to fractures—The Swedish Adherence Register Analysis (SARA). Osteoporos. Int..

[7] Laius O., Pisarev H., Maasalu K., Kõks S., Märtson A. (2017). Adherence to osteoporosis medicines in Estonia—A comprehensive 15-year retrospective prescriptions database study. Arch. Osteoporos..

[8] Zelko E., Klemenc-Ketis Z., Tusek-Bunc K. (2016). Medication adherence in elderly with polypharmacy living at home: A systematic review of existing studies. Mater. Sociomed..

[9] Pasina L., Brucato A.L., Falcone C., Cucchi E., Bresciani A., Sottocorno M., Taddei G.C., Casati M., Franchi C., Djade C.D. (2014). Medication non-adherence among elderly patients newly discharged and receiving polypharmacy. Drug. Aging.

[10] Rajpura J., Nayak R. (2014). Medication adherence in a sample of elderly suffering from hypertension: Evaluating the influence of illness perceptions, treatment beliefs, and illness burden. J. Manag. Care Pharm..

[11] Lee S.K., Kang B.Y., Kim H.G., Son Y.J. (2013). Predictors of medication adherence in elderly patients with chronic diseases using support vector machine models. Healthc. Inform. Res..

[12] Lee V.W., Pang K.K., Hui K.C., Kwok J.C., Leung S.L., Yu D.S., Lee D.T. (2013). Medication adherence: Is it a hidden drug-related problem in hidden elderly?. Geriatr. Gerontol. Int..

[13] Henriques M.A., Costa M.A., Cabrita J. (2012). Adherence and medication management by the elderly. J. Clin. Nurs..

[14] Gellad W.F., Grenard J.L., Marcum Z.A. (2011). A systematic review of barriers to medication adherence in the elderly: Looking beyond cost and regimen complexity. Am. J. Geriatr. Pharmacother..

[15] Park K.A., Kim J.G., Kim B.W., Kam S., Kim K.Y., Ha S.W., Hyun S.T. (2010). Factors that Affect Medication Adherence in Elderly Patients with Diabetes Mellitus. Korean Diabetes J..

[16] Smith H., Hankins M., Hodson A., George C. (2010). Measuring the adherence to medication of elderly patients with heart failure: Is there a gold standard?. Int. J. Cardiol..

[17] Raehl C.L., Bond C.A., Woods T.J., Patry R.A., Sleeper R.B. (2006). Screening tests for intended medication adherence among the elderly. Ann. Pharmacother..

[18] MacLaughlin E.J., Raehl C.L., Treadway A.K., Sterling T.L., Zoller D.P., Bond C.A. (2005). Assessing medication adherence in the elderly: Which tools to use in clinical practice?. Drug. Aging.

[19] Hayes T.L., Larimer N., Adami A., Kaye J.A. (2009). Medication Adherence in Healthy Elders. J. Aging Health.

[20] Lewiecki E.M. (2010). Bisphosphonates for the treatment of osteoporosis: Insights for clinicians. Ther. Adv. Chronic. Dis..

[21] Marcum Z.A., Gellad W.F. (2012). Medication Adherence to Multidrug Regimens. Clin. Geriatr. Med..

[22] Haugbølle L.S., Herborg H. (2009). Adherence to treatment: Practice, education and research in Danish community pharmacy. Pharm. Pract..

[23] Huber C.A., Reich O. (2016). Medication adherence in patients with diabetes mellitus: Does physician drug dispensing enhance quality of care? Evidence from a large health claims database in Switzerland. Patient Prefer Adherence.

[24] Hallgren J., Fransson E.I., Reynolds C.A., Finkel D., Pedersen N.L., Dahl Aslan A.K. (2018). Cognitive trajectories in relation to hospitalization among older Swedish adults. Arch. Gerontol. Geriatr..

